# A Case of Swyer-James-Macleod Syndrome Associated with Middle Lobe Hypoplasia and Arteriovenous Malformation

**DOI:** 10.1155/2012/959153

**Published:** 2012-12-09

**Authors:** Hatice Kaplanoglu, Veysel Kaplanoglu, Ugur Toprak, Alper Dilli, Baki Hekimoglu

**Affiliations:** ^1^Department of Radiology, Diskapi Yildirim Beyazit Training and Research Hospital, Ministry of Health, 06110 Ankara, Turkey; ^2^Department of Radiology, Ankara Numune Training and Research Hospital, 06410 Ankara, Turkey

## Abstract

A 58-year-old female patient presented to the hospital with hearing loss. In the chest radiography obtained before her ear surgery, volume decrease in the right hemithorax, elevation of the right diaphragm, and increase of ventilation in the right lung were detected. At the thorax CT-CT angiography, hypoplasia of the main pulmonary artery and its branches and arteriovenous malformation localized in the middle lobe of the right lung were detected. Thus, diagnosis of Swyer-James-Macleod syndrome associated with right lung middle lobe hypoplasia and arteriovenous malformation was made. This kind of association has not been reported earlier, so we are presenting it in the light of the literature knowledge.

## 1. Introduction

Swyer-James-Macleod, or single-sided clear lung syndrome (SJMS), frequently occurs in infancy and in childhood after adenovirus infections. It is a rare syndrome developing secondarily to bronchiolitis obliterans [1]. 

For the first time, this syndrome was defined by Swyer and James in 1953 in a 6-year-old boy with unilateral emphysema treated with pneumonectomy. A year later, Macleod reported a series of 9 cases with unilateral hyperlucency. From this date on, the disease was defined as Swyer-James-Macleod syndrome (SJMS) [1].

Most of the cases are asymptomatic. They are detected accidentally in chest radiographies obtained in adults. The characteristic radiologic findings are unilateral air trapping, hyperlucency signs, small or normal sized lungs, and decrease in the number and dimensions of the pulmonary vessels [1].

We present this study because no earlier case of Swyer-James-Macleod syndrome associated with middle lobe hypoplasia of the right lung and arteriovenous malformation has been presented.

## 2. Case

A fifty-eight-year-old female patient presented to the hospital with hearing loss. At the physical examination, conductive hearing loss of the right ear was detected. On the physical examination prior to the ear surgery, chest expansion, sounds, and reduced respiratory action of the right hemithorax. The left hemithorax was found to be normal. All other systems were normal on examination. When the patient was requisitioned from this point, it was found out that she has had shortness of breath since childhood. She has presented with a 3-year history of progressive exertional dyspnea and she has complained of productive cough with scant amounts of mucoid sputum and infrequent retrosternal chest pain within the recent two years. She has been taking asthma treatment since 3 years. Chest pain was unrelated to physical activity or emotional stress.

She denied any history of pulmonary infections such as measles, pertussis, recurrent pulmonary infection, bronchitis, or pneumonitis during childhood. She had no family history of pulmonary diseases. Her medical history revealed hypertension and stroke 12 years ago. On physical examination, the blood pressure was 136/38 mmHg, the pulse rate 67/min and regular, and respiratory rate 22/min. On laboratory tests, hemoglobin: 12.2 g/dL, hematocrit: %32.9, K: 3.48 (3.6–4.8) mmol/L, Ca: 1.09 (1.15–1.35) mmol/L, Cl: (95–100)104 mmol/L, and CK: 27 (32–294) U/L. Other blood and biochemistry levels were normal. Room air arterial blood gas analysis were as follows: O_2_ saturation %84, *P*_O_2__: 44.9 mmHg, *P*_CO_2__: 52.7 mmHg, pH: 7.35 (7.37–7.45), and bicarbonates 28.8 mmol/L. The total lung capacity %127, residual volume %194, ratio of residual volume to total lung capacity %47 and carbon monoxide diffusing capacity %122, forced vital capacity (FVC): %55.1, forced expiratory volume at first second (FEV1): %53,5, and Tiffeneau index (FEV1/FVC): %82,2. Respiratory function tests showed mild obstructive type disorder.

**Figure 1:**
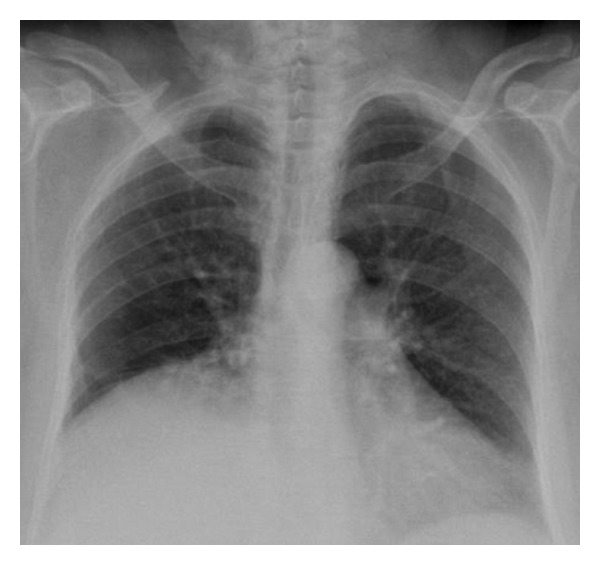
On the PA chest radiography, the volume of the right hemithorax was decreased and the hemidiaphragm elevated. The right pulmonary hilus was small, and hyperlucency and decrease of peripheral vascular shadowing of the right lung were detected.

**Figure 2:**
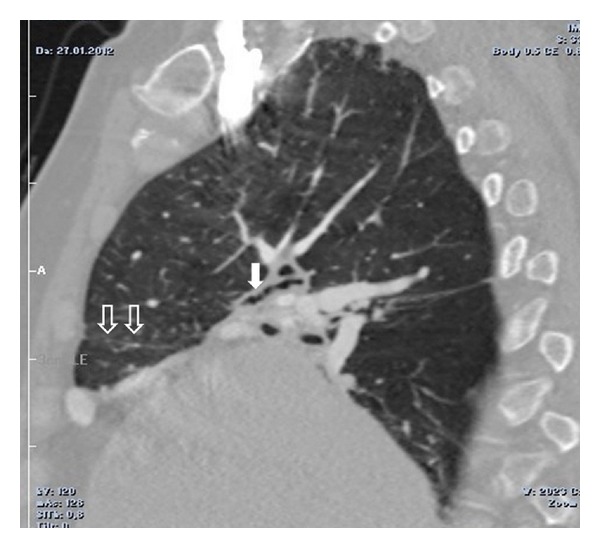
On sagittal CT image, hypoplasia of the right intermediate and middle lobe bronchus (white arrow), parenchymal scars and loss of ventilation in the middle lobe of the right lung were seen (open arrows).

**Figure 3:**
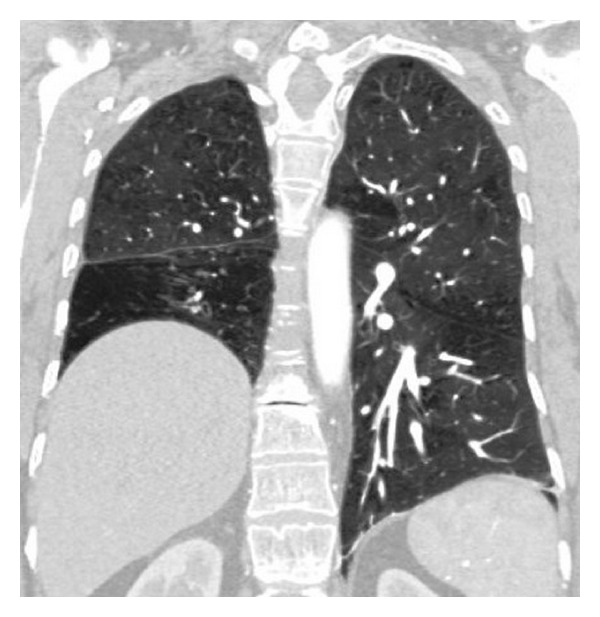
On coronal CT image, emphysema and parenchymal scars were seen in the lower lobe of the right lung.

**Figure 4:**
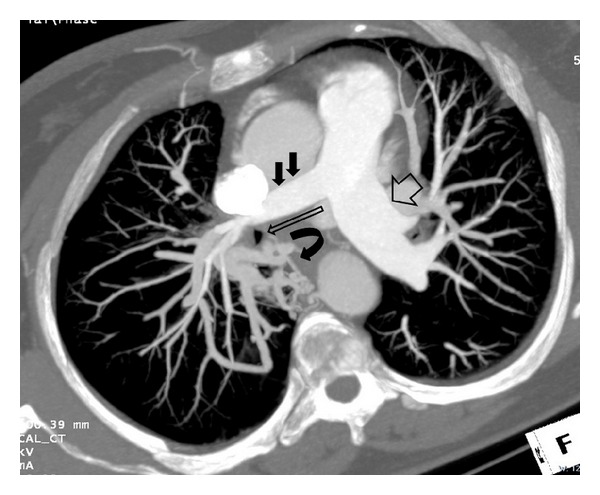
On Axial MIP CT image; at the upper and lower branches of the right main pulmonary artery (black arrows) diffuse hypoplasia, left main pulmonary artery (open thick arrow), hypoplasia of the right intermediate and middle lobe bronchus (open long arrow) was detected.

**Figure 5:**
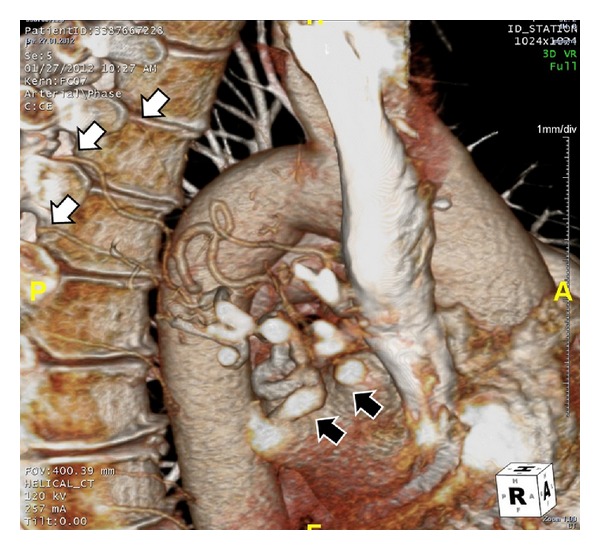
On 3D CT image, AVM was seen feeding from bronchial arteries (white arrows), draining to right pulmonary vein (black arrows).

 On the PA chest radiography, the volume of the right hemithorax was decreased and the hemidiaphragm elevated. The right pulmonary hilus was small and hyperlucency and decrease of peripheral vascular shadowing of the right lung were detected (Figure 1). Thorax CT and CT angiography were obtained. The minor fissure of the right lung was not observed. Hypoplasia and loss of ventilation in the middle lobe of the right lung and hypoplasia of the right intermediate and middle lobe bronchus were detected (Figure 2). Emphysema, parenchymal scars, and peribronchial thickening of the lower lobe bronchi were seen in the lower lobe of the right lung (Figure 3). At the upper and lower branches of the right main pulmonary artery, diffuse hypoplasia was detected (Figure 4). Various veins were observed starting from the right pulmonary cardiophrenic recess continuing through the surface of the diaphragm to the thoracic wall. On 3D CT image, AVM was seen feeding from bronchial arteries (white arrows), draining to right pulmonary vein (black arrows) (Figure 5). And this was defined as arteriovenous malformation.

On fiberoptic bronchoscopy, no endobronchial lesion was found. The electrocardiography was in normal sinus rhythm and normal axis and the echocardiography was also normal. There was no history of Rendu Osler Weber (ROW) syndrome in the family.

In accordance with the radiologic findings, the patient history, and other clinical data, diagnosis of Swyer-James-Macleod syndrome associated with right middle lobe hypoplasia and arteriovenous malformation was made. Bronchodilators were prescribed as therapy. Influenza and pneumococcus vaccines were administered and the patient was discharged with a periodic follow-up recommendation.

## 3. Discussion

Swyer-James-Macleod or single-sided hyperlucent lung is a rare syndrome often developed in infancy or childhood after adenovirus infection due to bronchiolitis obliterans [1]. Pulmonary artery agenesis and/or hypoplasia is characterized with pulmonary parenchyma hypoperfusion and the typical radiologic finding is single-sided translucent or hyperlucent lung [2]. In a study involving 17.450 chest radiographies, the ratio of the rarely encountered Swyer-James-Macleod syndrome was 0.001% [3]. 

Bronchiolitis obliterans results with inflammation of the respiratory bronchiole wall, fibrosis, and luminal narrowing. The fibrosis, in the intra-alveolar septa results with obliteration of the pulmonary capillary bed resulting in blood flow decrease in the main pulmonary artery segments. As a result, hypoplastic arterial formation occurs [4]. The decrease of the compensatory perfusion in the peripheral respiratory airways develop. These pathophysiologic changes lead to air trapping and hypoperfusion in the affected segment, thus creating radiographic hyperlucent or translucent findings [4].

Clinically, most patients are asymptomatic. Patients with this syndrome have been described with dyspnea, cough, hemoptysis, recurrent pulmonary infection, and failure to thrive [5, 6]. The disease may not be diagnosed until adulthood. As in the case presented, other cases are diagnosed accidentally when obtaining chest radiography for other reasons [1, 6]. The presented case had shortness of breath since childhood and diagnosis of asthma 3 years since. Remaining asymptomatic until this late age might be due to not developing bronchiectasis or being localized in the right lower lobe. SJMS can affect one or more pulmonary lobes or one or more segments of a lobe. However, very rarely may be bilateral [7].

The diagnosis of SJMS is based on radiological findings. Classical chest radiography finding is unilateral lobar single-sided hyperlucency due to oligemia. This appearance is similar to mosaic pattern [7]. The presented case had similar chest radiography. Bronchoscopy might be needed in the differential diagnosis of intrabronchial benign or malignant neoplastic obstruction, bronchial atresia, pulmonary artery stenosis, and less frequent diseases like pulmonary embolism making single-sided hyperlucency [8]. At the presented case, bronchoscopy was performed in order of ruling out other causes of hyperlucency. In the respiratory function tests of SJMS mild to moderate obstructive type disorders are generally present as it was in our case [3].

CT plays an important role in SJMS as it can detect parenchymal damage and show pulmonary vascular distribution and bronchiectasia [6]. Characteristically in CT angiography, pulmonary artery hypoplasia/agenesis and decrase of pulmonary artery calibration can be detected, thus decrease of pulmonary blood suply can be detected. Other characteristic findings are pathologic perfusion changes at the affected lung, heterogenous patchlike air trapping, and atelectasis of healthy pulmonary areas due to compression [9]. For this reason, the disease is better diagnosed with high resolution CT and CT angiography. 

Ventilation perfusion scintigraphy can also be performed, but in the presence of distal respiratory tract diseases (asthma, congenital lobar emphysema) may give false positive results [10]. Although usually in the affected side of SJMS bronchiectasis is present, in the presented case, symptoms, fisical examination findings, or CT findings of bronchiectasis were absent.

Pulmonary arteriovenous malformations (PAVMs) are very uncommon pathologies of unknown etiology. Children constitute 10% of the cases and the incidence increases at the fifth-sixth decade. The syndrome most commonly associated with congenital AVMs is ROW syndrome. The transition in question is heritable, characterized with recurrent epistaxis and mucocutaneous telangiectasia. AVM's can also be seen in extrapulmonary organs and usually more than one. 

On contrary in isolated PAVMs, there is no hereditary transition and no extrapulmonary lesions [10]. PAVM's are more common in females, mostly in lower lobes and unilaterally. Half of the cases are nourished from the systematic circulation and 80% from a single artery. Venous drainage is systemic or to the pulmonary veins [10]. Usually it is nourished from the afferent branches of the pulmonary artery but it can be nourished directly from bronchial arteries, intercostal arteries, or the aorta. Venous drainage is often to the pulmonary vein and rarely directly to the left atrium [11]. Despite of being rare in the case presented, the malformation was among the bronchial artery and the pulmonary vein. 

The symptoms in PAVM's change according to the number and the dimensions of the lesion. While the single lesions smaller than 2 cm are usually asymptomatic, the bigger lesions can present with symptoms as coughing, shortness of breath, haemoptysis, cyanosis, and epistaxis [12]. In our case, the AVM's were smaller than 2 cm and asymptomatic. But the patient had history of stroke. The AVM's usually encountered in the clinics are single, asymptomatic, and accidentally detected in routine chest radiographies [13]. PAVM's can be visualized with pulmonary angiography and magnetic resonance (MR) angiography. But nowadays CT angiography is as least sensitive and specific as pulmonary angiography. Pulmonary angiography is recommended in cases planed for transcatheter embolization [14].

In most of the SJMS case reports, the hemithorax is entirely affected. In previous case reports, hyperlucency associated with lobar anomaly was reported in one case only [15]. Local hyperlucency as in the case presented is rare. SJMS characterized with local hyperlucency associated with middle lobe hypoplasia and AVM makes the case even more rare. It is also of importance the asymptomatic nature of these patologies until the age of 58 is also of importance. For these reasons, we should consider that we might come across these patients in their late age.
